# Draft Genome Sequence of Tolypothrix boutellei Strain VB521301

**DOI:** 10.1128/genomeA.00001-15

**Published:** 2015-02-19

**Authors:** Mathu Malar Chandrababunaidu, Deeksha Singh, Diya Sen, Sushma Bhan, Subhadeep Das, Akash Gupta, Siba Prasad Adhikary, Sucheta Tripathy

**Affiliations:** a Structural Biology and Bioinformatics Division, Council of Scientific and Industrial Research, Indian Institute of Chemical Biology, Kolkata, West Bengal, India; b Fakir Mohan University, Vyasa Vihar, Nuapadhi, Balasore, Odisha, India

## Abstract

We report here the draft genome sequence of the filamentous nitrogen-fixing cyanobacterium Tolypothrix boutellei strain VB521301. The organism is lipid rich and hydrophobic and produces polyunsaturated fatty acids which can be harnessed for industrial purpose. The draft genome sequence assembled into 11,572,263 bp with 70 scaffolds and 7,777 protein coding genes.

## GENOME ANNOUNCEMENT

Cyanobacteria are photosynthetic prokaryotes producing a variety of compounds with applications ranging from industrial products, health care, and food biotechnology to environmental remediation (1). They are also known to produce bioactive compounds with antimicrobial (2) antifungal properties (3) and are implicated in the production of biofuel (4) and biodegradable plastics (5, 6).

Tolypothrix is a filamentous, hydrophobic nitrogen-fixing cyanobacterium with great commercial significance (7). Some members of this genus have been found to produce antifungal glycopeptides (8) and blood-clotting agents (9). Fatty acid profiling of Tolypothrix spp. has shown high oleic acid content and the monounsaturated fatty acid produced by these organisms can be used as biodiesel (10).

Tolypothrix boutellei strain VB521301 was isolated from stone monuments in Shantiniketan, India (11). Cultures were maintained in BG11 medium at 26°C under a 16/8-h light/dark cycle. The cultures were shaken once daily to untangle filaments. Colonies on solid BG11 slants appeared as clumps of filaments and the liquid cultures were dark green in color.

Genomic DNA was isolated and purified using a Uniflex Bacterial DNA isolation kit (Genei, USA). A total of 1.86 µg of genomic DNA with a concentration of 143.08 ng/µl was used for sequencing. Whole-genome sequencing was carried out on an Illumina Hiseq platform.

A paired-end library was constructed with ~300 bp insert size and sequenced at 90.0× coverage generating approximately 7.4 million reads and 151 bases read length. A second mate-pair library was constructed with a 3 kb insert size and sequenced at 35× coverage generating 6 million reads and an average read length of 101 bases. Initially the raw reads of both mate-pair and paired-end libraries were assembled by using the A5 pipeline (12). The contigs from the first assembly were randomly sheared generating 6 k and 20 k long jump libraries, 101 bases long at 10× coverage using a simulated read generator wgsim (13). We did a final assembly using the paired-end and mate-pair data (3-kb, 6-kb, and 20-kb libraries) with Allpaths-LG-49856 (14). The final assembly produced a genome of 11.5 MB with 70 scaffolds and an *N*_50_ score of 1,987,087 bases. The length of the largest and smallest scaffolds was 2,493,347 and 2,000 bases, respectively, and the G+C content was calculated to be 42%. The sequences were submitted to GenBank and annotated using the PGAP pipeline (http://www.ncbi.nlm.nih.gov/genome/annotation_prok/). A total of 7,777 protein coding genes; 35 clustered regularly interspaced short palindromic repeats (CRISPR) genes; 6 rRNA genes; 92 tRNA genes; 1,275 pseudogenes, and 1 noncoding RNA (ncRNA) gene were predicted. We performed gene space analysis by searching for the presence of cyanobacterial specific core orthologs (15) and 378/384 were present in this assembly.

About 50% of the genes (3,258) have no known similarity to previously annotated genes. At least a dozen of the alcohol dehydrogenase genes are present in this genome. Several important genes such as VioC monooxygenase (coding for antibiotics) and CheY responsible for quorum sensing are predicted. Genome sequence and assembly of Tolypothrix boutellei VB521301 can be further elucidated for commercial exploitation.

### Nucleotide sequence accession number.

The Tolypothrix boutellei VB521301 genome sequence and annotation data have been deposited in GenBank under the accession no. JHEG00000000.
